# Complete Genome Sequences of Pseudomonas atacamensis Strain SM1 and Pseudomonas toyotomiensis Strain SM2, Isolated from the Date Palm Rhizosphere

**DOI:** 10.1128/MRA.00253-21

**Published:** 2021-05-06

**Authors:** Yassine Elmahi, Mohammed Saeed Alshamsi, Naganeeswaran Sudalaimuthuasari, Biduth Kundu, Raja S. AlMaskari, Khaled M. Hazzouri, Subha Chandran, Shahana Sehar Malik, Sunil Mundra, Khaled M. A. Amiri

**Affiliations:** a Department of Biology, College of Science, United Arab Emirates University, Al Ain, United Arab Emirates; b Khalifa Center for Genetic Engineering and Biotechnology, United Arab Emirates University, Al Ain, United Arab Emirates; Loyola University Chicago

## Abstract

Here, we announce the complete genome sequences of two phosphate-solubilizing rhizobacteria, Pseudomonas atacamensis strain SM1 (genome size, ∼5.9 Mb) and Pseudomonas toyotomiensis strain SM2 (genome size, ∼5.2 Mb), isolated from the rhizosphere of date palms growing in the oasis agroecosystem of the United Arab Emirates (UAE).

## ANNOUNCEMENT

The phosphate-solubilizing rhizobacteria Pseudomonas atacamensis strain SM1 and Pseudomonas toyotomiensis strain SM2 were isolated from the rhizosphere of date palms on a farm located in the emirate of Ras Al-Khaimah, United Arab Emirates (UAE) (25.8007°N, 55.9762°E). Rhizosphere samples were mixed thoroughly with distilled water (1:9 ratio) and inoculated into standard Pikovskaya’s agar medium to screen for phosphate-solubilizing bacteria. The inoculated plates were incubated at 30°C for 48 h. Two positive colonies (SM1 and SM2), which developed a clear zone around themselves, were selected and further purified in the same culture medium at 30°C for 48 h. DNA from the culture was isolated using an XpressDNA bacterial kit (MagGenome Technologies, Chennai, India).

Whole-genome sequencing (WGS) of both SM1 and SM2 were carried out using the Oxford Nanopore (long-read) and Illumina (short-read) platforms. Oxford Nanopore WGS libraries were prepared using a ligation sequencing kit (SQK-LSK 109) and sequenced using the Oxford Nanopore MinION system (flow cell, FLO-MIN106D R9.4 revision D chip). Illumina-compatible WGS libraries were prepared using the NEBNext Ultra II DNA library preparation kit and sequenced on an Illumina NovaSeq 6000 system (150-bp paired-end sequencing chemistry). Guppy v.3.3.2 software (implemented in the MinKNOW interface; Oxford Nanopore, Cambridge, UK) was used for base calling, demultiplexing, and adapter trimming of the MinION-generated reads.

The MinION sequencing produced 259,780 reads for SM1 (*N*_50_, 12,821 bp; coverage, 200×) and 220,922 reads for SM2 (*N*_50_, 7,260 bp; coverage, 120×). Sequencing errors found in the long reads were corrected using CANU v.1.8 (1) software with default parameters. After error correction, reads longer than 1,000 bp were considered for the genome assembly process. In total, 12,780,120 and 15,983,612 Illumina raw reads were obtained from the Illumina sequencing for Pseudomonas atacamensis strain SM1 and Pseudomonas toyotomiensis strain SM2. The quality of the raw reads was checked using FastQC (2), and the reads were trimmed for adapters and low-quality regions using Trimmomatic v.0.39 (3) (MINLEN:50 TOPHRED33). After adapter and quality trimming, 98% of reads were retained for both the SM1 and SM2 samples. We applied a hybrid *de novo* genome assembly approach (using both Illumina and MinION reads), and genome error correction (using the Illumina short reads) was performed using the SPAdes v.3.11.1 tool (4) with default settings. The quality and completeness of the genome assembly was confirmed using the BUSCO v.3 tool (5), while gene prediction and genome annotation were carried out using NCBI PGAP v.5.1 (6).

Genome assembly of Pseudomonas atacamensis strain SM1 resulted in a single circular genome comprising 5,909,586 bp (with a GC content of 60.1%) and a plasmid sequence (81,190 bp). Gene annotation of this genome resulted in 5,436 gene models (5,351 coding DNA sequences [CDSs], 13 rRNAs, 68 tRNAs, 4 noncoding RNAs [ncRNAs], and 35 pseudogenes). Genome assembly of Pseudomonas toyotomiensis strain SM2 resulted in a single circular genome comprising 5,235,458 bp (with a GC content of 62.7%). Gene annotation of the Pseudomonas toyotomiensis strain SM2 genome resulted in 4,857 gene models (4,776 CDSs, 12 rRNAs, 65 tRNAs, 4 ncRNAs, and 30 pseudogenes). The genome sequences of the above two phosphate-solubilizing bacterial strains will be useful for understanding their molecular mechanisms of promoting plant growth in an arid environment.

### Data availability.

The raw data (Illumina and Oxford Nanopore reads) generated during this experiment have been deposited in the NCBI SRA database under the BioProject accession numbers PRJNA701364 and PRJNA701365. The assembled genome and plasmid sequences were submitted to the NCBI GenBank database under the accession numbers CP070503 (SM1 genome), CP070504 (SM1 plasmid), and CP070505 (SM2 genome).
